# Supplementary material for: Superior zero thermal expansion dual-phase alloy via boron-migration mediated solid-state reaction
Source: Nat Commun. 2023 May 30;14:3135. doi: 10.1038/s41467-023-38929-0 (PMC10229566; doi:10.1038/s41467-023-38929-0)
Supplement: Supplementary file 1 — Supplementary Information [file 41467_2023_38929_MOESM1_ESM.pdf]

Supplementary Information for

Superior zero thermal expansion dual-phase alloy via boron-migration mediated solid-state reaction

Chengyi Yu[1], Kun Lin[1,*], Xin Chen[1], Suihe Jiang[1], Yili Cao[1], Wenjie li[1], Liang Chen[1], Ke An[2], Yan Chen[2], Dunji Yu[2], Kenichi Kato[3], Qinghua Zhang[4], Lin Gu[4], Li You[1], Xiaojun Kuang[5], Hui Wu[6], Qiang Li[1], Jinxia Deng[1] and Xianran Xing[1,*]

[1] Beijing Advanced Innovation Center for Materials Genome Engineering, and Institute of Solid State Chemistry, University of Science and Technology Beijing, Beijing 100083, China.

[2] Neutron Scattering Division, Oak Ridge National Laboratory, Oak Ridge, TN, USA.

[3] RIKEN SPring-8 Center, 1-1-1 Kouto, Sayo-Cho, Sayo-gun, Hyogo 679-5148, Japan.

[4] Beijing National Laboratory for Condensed Matter and Institute of Physics, Chinese Academy of Sciences, Beijing 100190, China

[5] Guangxi Key Laboratory of Electrochemical and Magnetochemical Functional Materials, College of Chemistry and Bioengineering, Guilin University of Technology, Guilin 541004, P. R. China.

[6] NIST Center for Neutron Research, National Institute of Standards and Technology, Gaithersburg, Maryland 20899-6102, United States.

*Corresponding author. Email: *xing@ustb.edu.cn*; *kunlin@ustb.edu.cn*

Contents

Supplementary Tables and Figures **4**

Supplementary Table 1 | The elemental content of Pre. Er-Fe-B alloy determined by EPMA.. .... **4**
Supplementary Table 2 | The long-range crystal structure information of $ErFe_{10}$ determined by SXRD. **5**
Supplementary Table 3 | The summary of compressive strength (δ_{CS}, MPa), Coefficient of thermal expansion (α, $\times 10^{-6}$ K^{-1}), and ZTE/NTE temperature windows in typical ZTE/NTE materials. **6**
Supplementary Fig. 1 | The microstructure of Pre. Er-Fe-B alloy (ZTE composition). **7**
Supplementary Fig. 2 | The elemental analysis by electro-probe micro-analyzer (EPMA). **8**
Supplementary Fig. 3 | The cryastal structure model of $ErFe_{10}$ phase........ **9**
Supplementary Fig. 4 | The Rietveld refinement of the Pre. Er-Fe-B alloy. **10**
Supplementary Fig. 5 | The TEM results of the $ErFe_{10}$ phase. **11**
Supplementary Fig. 6 | The crystal structure of the $ErFe_{10}$ phase is determined by single-crystal diffraction. **12**
Supplementary Fig. 7 | The structure model of the $ErFe_{10}$ phase. **13**
Supplementary Fig. 8 | The microstructural evolution (ZTE composition) in at different stages (0d, 1d, 3d, 4d and 5d). **14**
Supplementary Fig. 9 | The microstructure of Tar. Er-Fe-B alloy (ZTE sample). **15**
Supplementary Fig. 10 | Comparison of SXRD data between Pre. Er-Fe-B and Tar. Er-Fe-B alloy. **16**
Supplementary Fig. 11 | The microstructure of three different regions re-precipitated α phase. **17**
Supplementary Fig. 12 | The microstructure of three different regions re-precipitated α phase. **18**
Supplementary Fig. 13 | Lattice thermal expansion of the $Er_2Fe_{14}B$ compound........ **19**
Supplementary Fig. 14 | The Rietveld refinement pattern of the Tar. Er-Fe-B alloy at room temperature (λ = 0.45 Å). **20**
Supplementary Fig. 15 | The X-ray diffraction profiles.. .... **21**
Supplementary Fig. 16 | The comparison of compositions versus CTE and contents of α phase. **22**
Supplementary Fig. 17 | The magnetization curves of the Pre. Er-Fe-B (ZTE composition) and Tar. Er-Fe-B alloys........ **23**
Supplementary Fig. 18 | The microstructure of the Tar. Er-Fe-B after 200th thermal cycles. **24**
Supplementary Fig. 19 | The two typical phase interfaces of the Tar. Er-Fe-B alloy. **25**
Supplementary Fig. 20 | Schematic diagram of the real-time in-situ neutron diffraction experimental set-up from the top view. **26**
Supplementary Fig. 21 | The summary of compressive strength versus temperature window of typical ZTE alloys........ **27**
Supplementary Fig. 22 | The in-situ loading neutron diffraction patterns of the Tar. Er-Fe-B were collected by Bank 1 and bank 2 probes, respectively........ **28**
Supplementary Fig. 23 | Lattice evolution of the E and α phase as a function of applied stress determined by full pattern Rietveld refinement. **29**

Supplementary Fig. 24 | The microstructure of the fracture surface. a The SEM image of the fracture surface.**.......... 30**
Supplementary Fig. 25 | All possible chemical configurations of this hexagonal lattice.**.. 31**
Supplementary References.......... 32

Supplementary Tables and Figures

Supplementary Table 1 | The elemental content of Pre. Er-Fe-B alloy determined by EPMA. The corresponding area is marked in Supplementary Fig. 2 (red circles).

**Areas**	**Er, atom. %**	**Fe, atom. %**	**Ratio**	**Phase**
1	9.35	90.65	1:9.70	$ErFe_{10}$
2	0.24	99.76	1:415.67	α-Fe (FeB_x)
3	12.54	87.46	2:13.95	$Er_2Fe_{14}B$

Note: The atomic number of boron is too low to be accurately detected.

Supplementary Table 2 | The long-range crystal structure information of $ErFe_{10}$ determined by SXRD.

Phase	Atoms	*x*	*y*	*z*	Occ.	Site	Sym.
	Er_1	0.00000	0.00000	0.25000	0.772	2*b*	-6*m*2
	Er_2	0.33333	0.66667	0.75000	1.000	2*d*	-6*m*2
	Fe_1	0.33333	0.66667	0.10676	1.000	4*f*	3*m*.
$ErFe_{10}$	Fe_2	0.50000	0.00000	0.00000	1.000	6*g*	.2/*m*.
	Fe_3	0.33415	0.95614	0.25000	1.000	12*j*	*m*..
	Fe_4	0.16667	0.33296	0.97899	1.000	12*j*	.*m*.
	Fe_5	0.00000	0.00000	0.89400	0.231	4*e*	3*m*.

Supplementary Table 3 | The summary of compressive strength (δ_{CS}, MPa), Coefficient of thermal expansion (α, $\times10^{-6}$ K^{-1}), and ZTE/NTE temperature windows in typical ZTE/NTE materials.

Typical ZTE materials	Compressive strength (δ_{CS}, MPa)	Coefficient of thermal expansion (CTE, $\times10^{-6}$ K^{-1})	Temperature Windows (ΔT, K)	Refer.
$TbCo_{1.9}Fe_{0.1}$	260	0.48 (α_l)	123−307 (ΔT=184)	1
$Ho_2Fe_{16}Cr$	50	1.3 (α_V)	13-330 (ΔT=317)	2
$La(Fe_{0.92}Co_{0.08})_{10.3}Al_{2.7}$	256.4	0.76 (α_l)	150-296 (ΔT=146)	3
LFCS/$Cu_{39.7\ vol.}$	360.6	0.2 (α_l)	200-320 (ΔT=120)	4
$LaFe_{11}Si_2H_x$	60	0.5 (α_a)	20-275 (ΔT = 255)	5
$Mn_3Cu_{0.5}Ge_{0.5}N$	161	0.12 (α_a)	12-230 (ΔT = 218)	6
$Ho_{0.04}Fe_{0.96}$	878	0.19 (α_l)	100-335 (ΔT =235)	7
GDCF	154	0.16 (α_a)	10-275 (ΔT =265)	8
GHCF	172	-0.91 (α_V)	10-275 (ΔT =265)	9
$LaFe_{18.2}Co_{1.2}Si_{1.6}$	968.8	-0.65 (α_l)	261-282 (ΔT =21)	10
$LaFe_{10.1}Cu_{0.5}Si_{2.4}$	1325	0.28 (α_l)	185-250 (ΔT =60)	11
$Ho_2Fe_{16}Co$	49	0.07 (α_l)	3-461 (ΔT=458)	12
Er-Fe-V-Mo	1631.4	1.87 (α_l)	100-493 (ΔT=393)	13
$La(Fe, Co, Si)_{13}$	256.4	-26.1 (α_l)	240-350 (ΔT=110)	14
Mn_3Ge	204	-7.58 (α_l)	297-374 (ΔT=77)	15
$Hf_{0.87}Ta_{0.13}Fe_2$	380	-16.3 (α_l)	222-327 (ΔT=105)	16
$Er_2Fe_{13.3}Co_{0.7}B$	191.4	1.5 (α_V)	120-475 (ΔT=355)	17
$MnCoGe_{0.99}In_{0.01}$	70.4	-94.7 (α_l)	192-310 (ΔT=108)	18
Tar. Er-Fe-B	1440	0.28 (α_l)	100-550 (ΔT=450)	**This work**

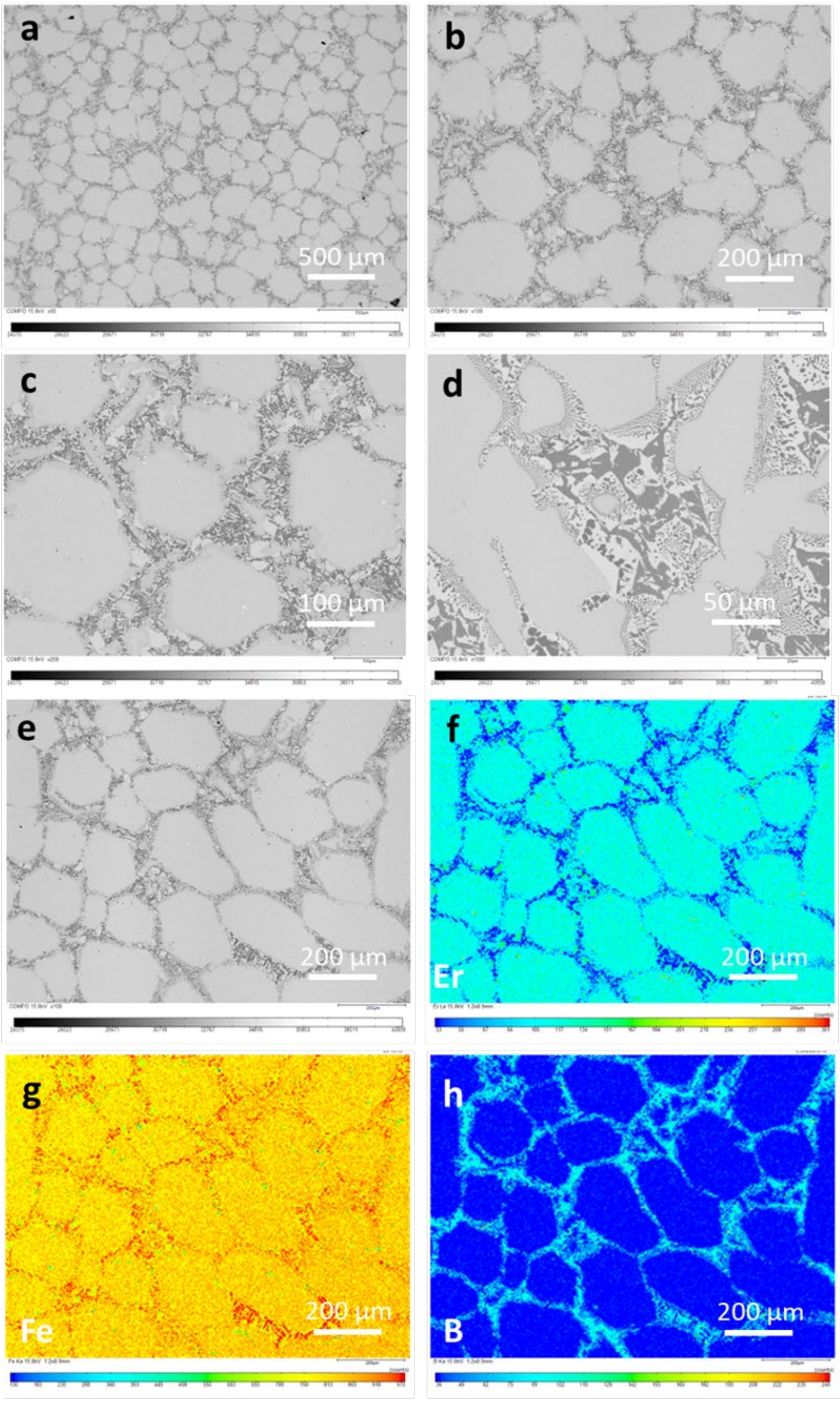

Supplementary Fig. 1 | The microstructure of Pre. Er-Fe-B alloy (ZTE composition). a-d The microstructure of Pre. Er-Fe-B at different scales. **e-f** The phase-contrast image (**e**) and corresponding elements mapping, Er (**f**), Fe (**g**), and B (**h**), respectively. The elements mapping results indicate the B atoms diffuse into the primary α phase at high temperatures.

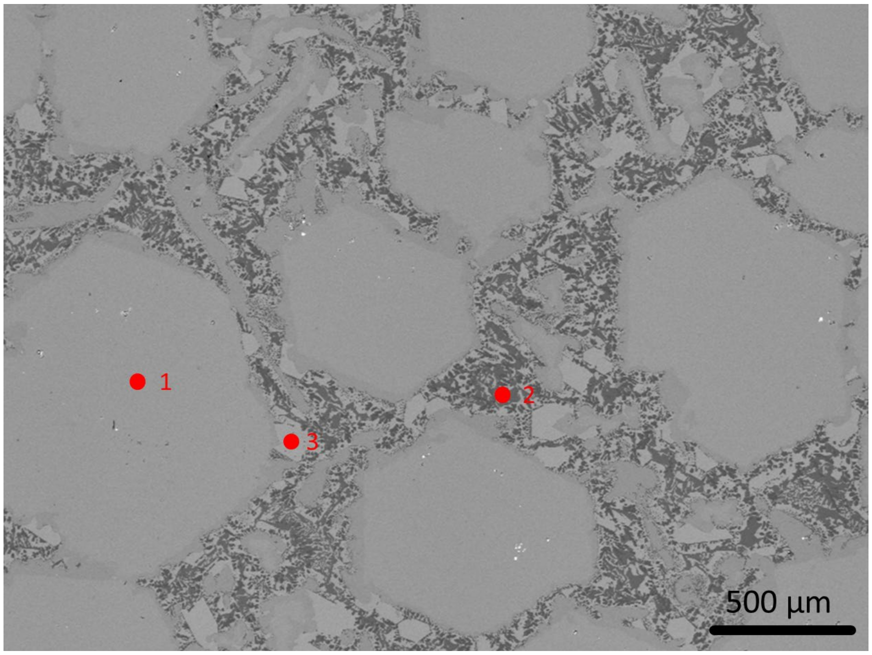

Supplementary Fig. 2 | The elemental analysis by electro-probe micro-analyzer (EPMA).

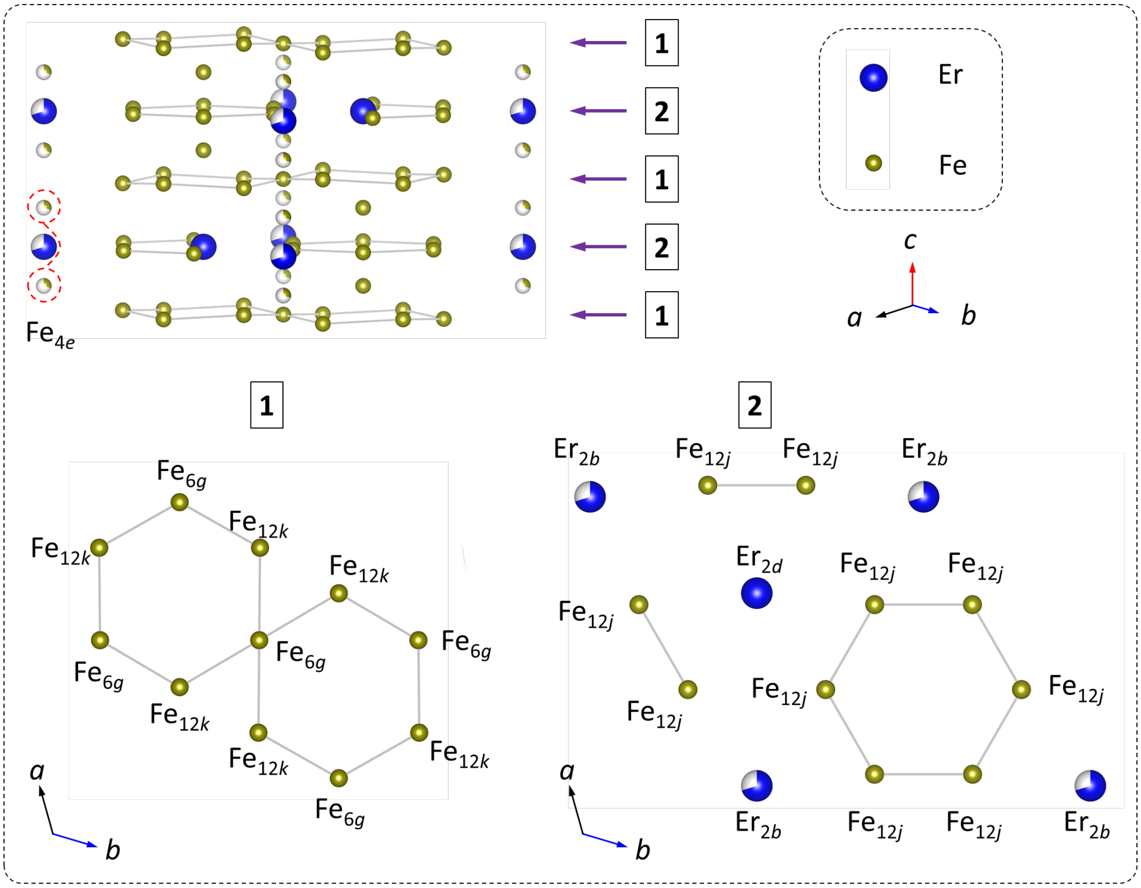

Supplementary Fig. 3 | The crystal structure model of the $ErFe_{10}$ phase.

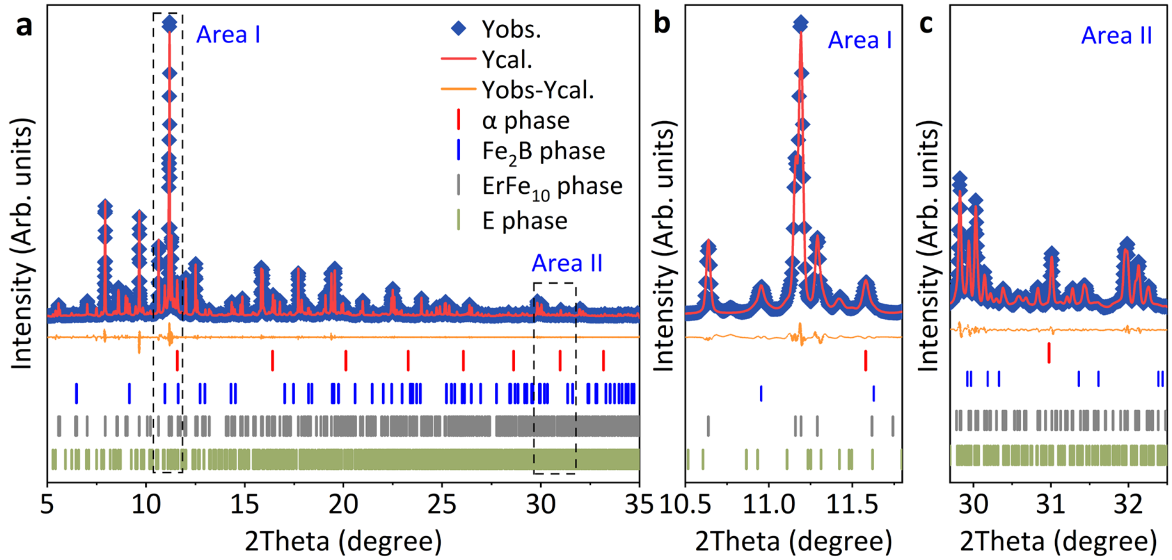

Supplementary Fig. 4 | The Rietveld refinement of the Pre. Er-Fe-B alloy. a The Rietveld refinement of the Pre. Er-Fe-B alloy. **b-c** The enlarged profiles are marked in (**a**). The synchrotron X-ray diffraction (λ= 0.4098 Å) confirmed the crystal structure of $ErFe_{10}$ is Er_2Fe_{17}-type hexagonal crystal symmetry (space group: $P6_3/mmc$). Another obvious B-rich phase at the grain boundary is a composite with α phase, Fe_2B, and little $Er_2Fe_{14}B$. We can attain a suitable fitting result by introducing Er_2Fe_{17}, α phase, Fe_2B, and $Er_2Fe_{14}B$ phases. And the Fe-B phases diagram further confirmed our results[19].

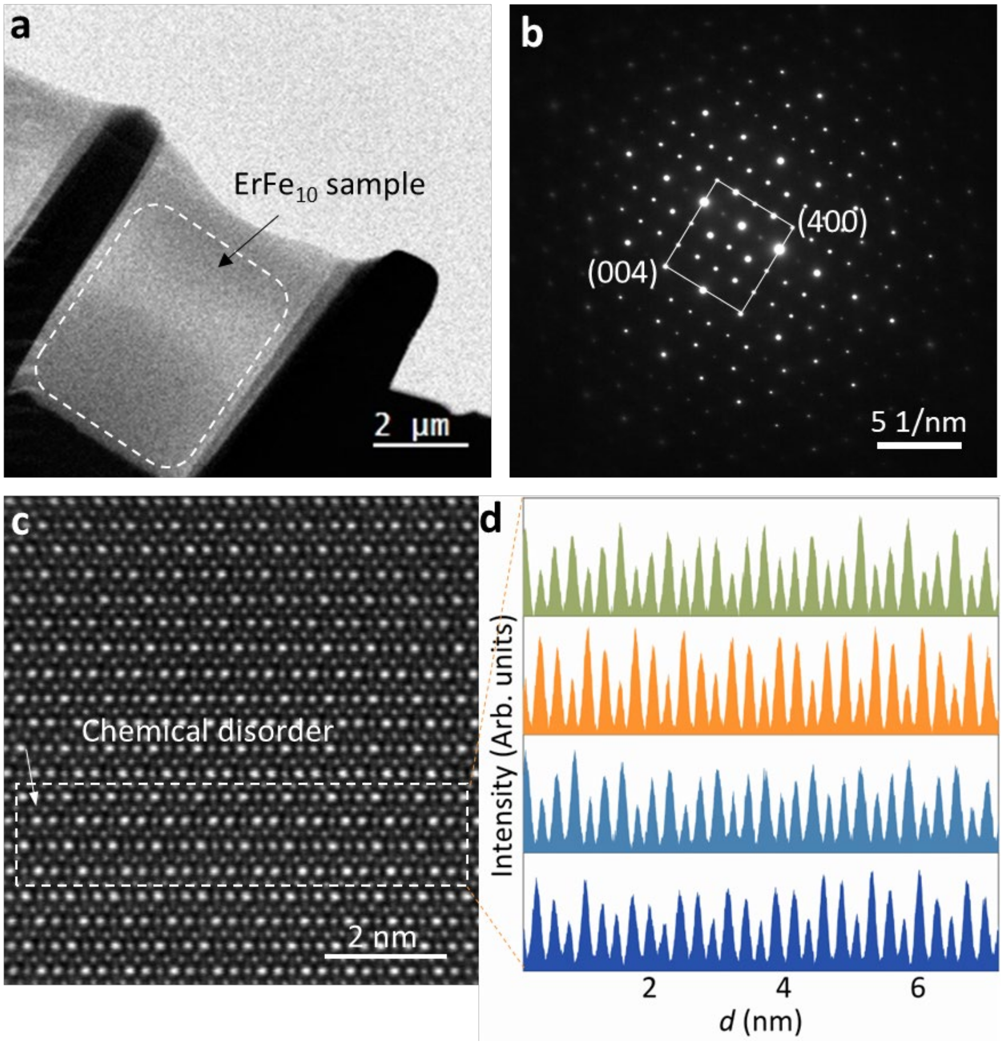

Supplementary Fig. 5 | The TEM results of the $ErFe_{10}$ phase. a The microstructure of the $ErFe_{10}$ sample. **b** The SAED of the HAADF-STEM images in (**c**). **c** The HADDF-STEM images of the $ErFe_{10}$ phase. **d** The intensity profile of the lattice is marked with a white rectangular in (**c**). The sample is fabricated by a focused-ion beam (FIB). The selected area electron diffraction (SAED) further confirmed hexagonal crystal symmetry (space group: $P6_3/mmc$). The random intensity profile of the lattice reveals long-range chemical disordering[20].

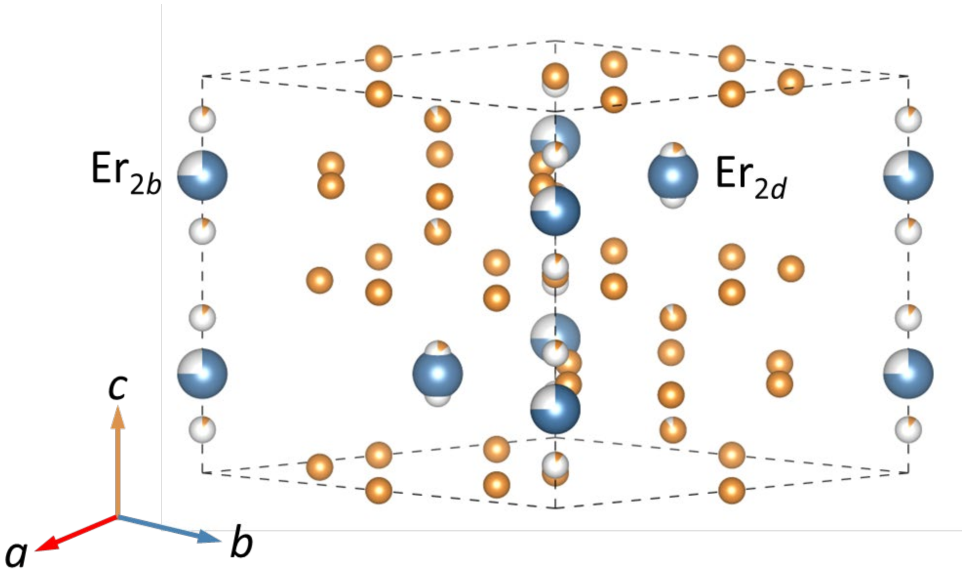

Supplementary Fig. 6 | The crystal structure of the $ErFe_{10}$ phase is determined by single-crystal diffraction. The single-crystal diffraction is conducted by artificially selecting a single crystal. The results also confirmed the Er_2Fe_{17}-type crystal structure. Due to the excess of the Fe atoms, the Wyckoff positions (WF) Er_{2b} site is displaced by Fe atoms disordered in the form of Fe-Fe pairs. Besides, The Wyckoff positions (WF) Er_{2d} site also observed substitutions by single Fe atoms and the Fe atoms deviate from the central WF site.

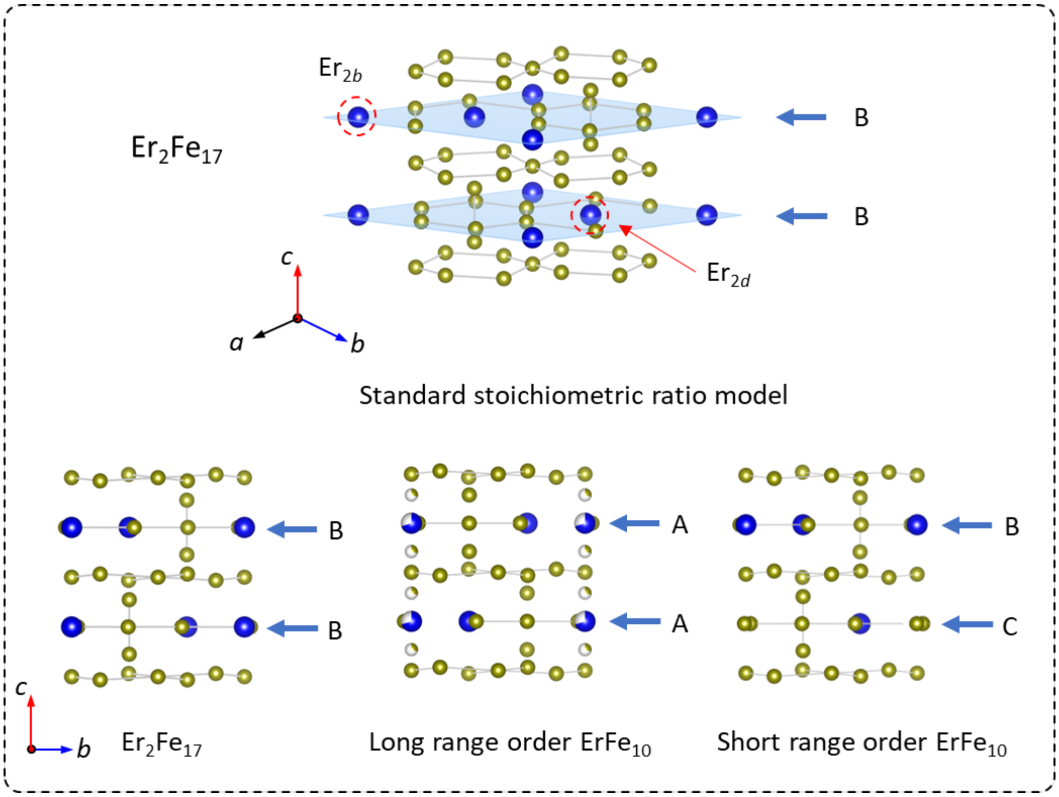

Supplementary Fig. 7 | The structure model of the Er_2Fe_{17} and $ErFe_{10}$ phases observed under STEM.

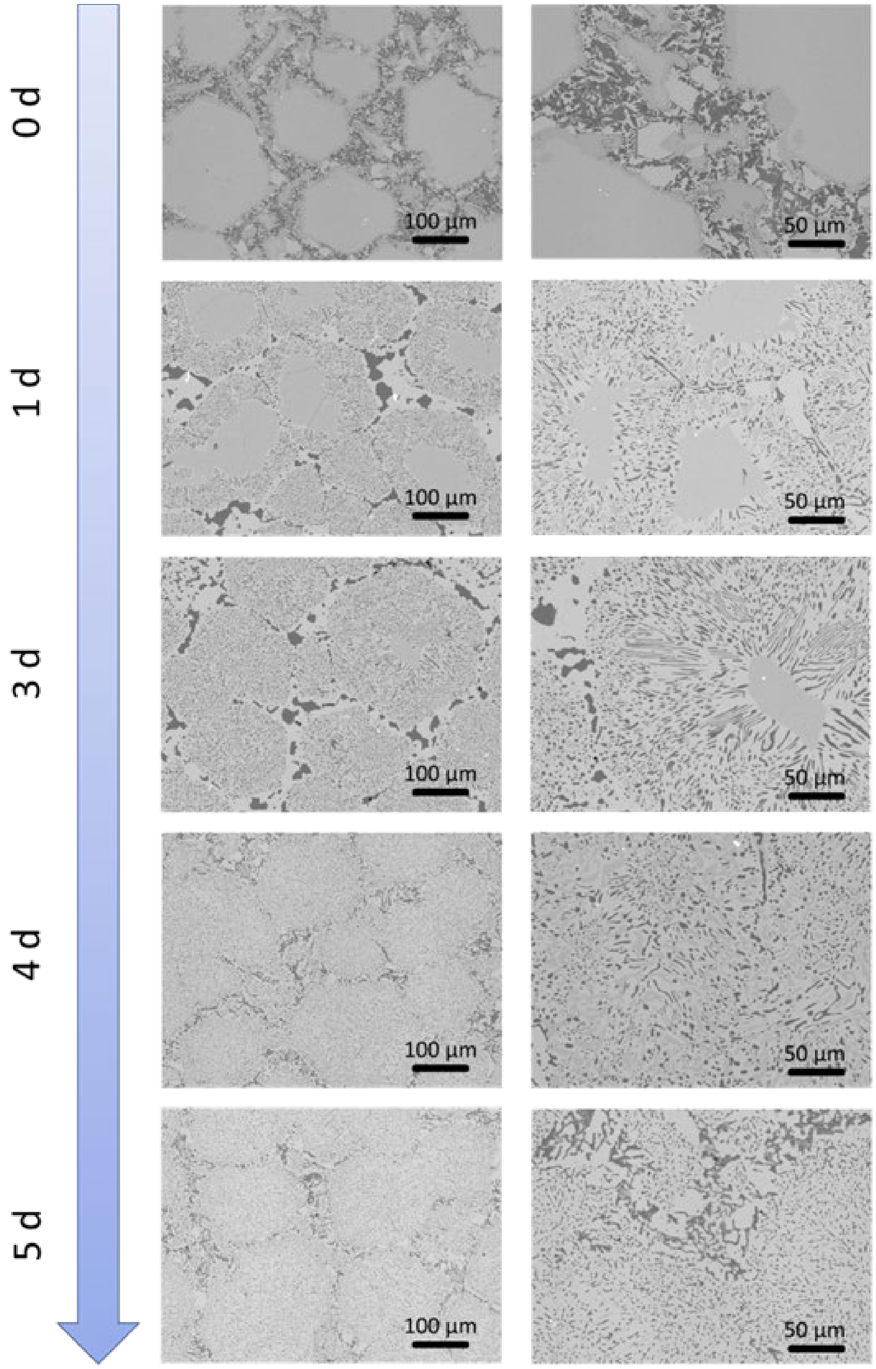

Supplementary Fig. 8 | The microstructural evolution (ZTE composition) at different stages (0d, 1d, 3d, 4d, and 5d).

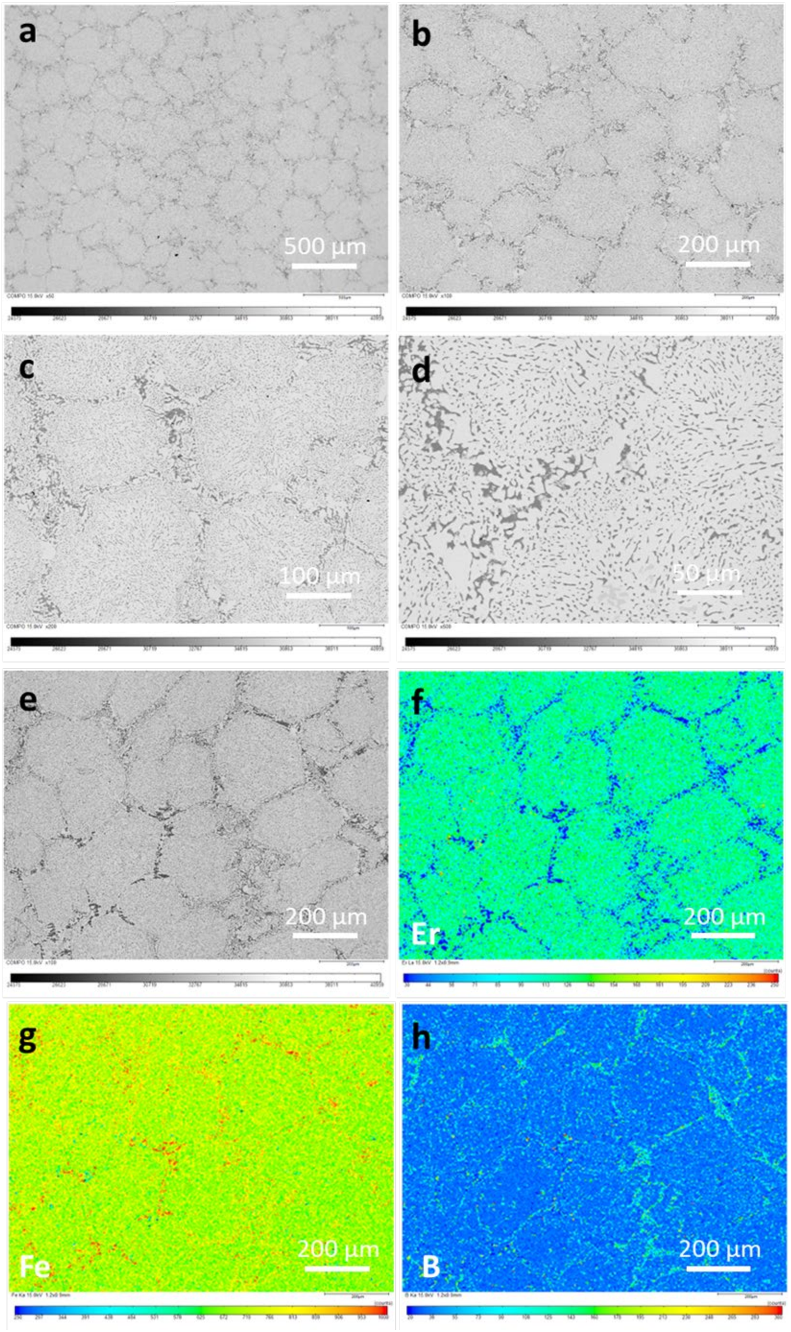

Supplementary Fig. 9 | The microstructure of Tar. Er-Fe-B alloy (ZTE sample). a-d The microstructure of Pre. Er-Fe-B at different scales. **e-h** The phase-contrast image (**e**) and corresponding elements mapping, Er (**f**), Fe (**g**), and B (**h**), respectively. The Tar. Er-Fe-B alloy is synthesized by the boron-migration-mediated solid-state reaction of the Pre. Er-Fe-B alloy. The re-precipitated α phase is homogeneously dispersed into the intragranular grains. According to the mapping of the elements, the B atoms diffused back to the matrix after the reaction. Besides, α phase parts of B atoms in dissolved in the α phase.

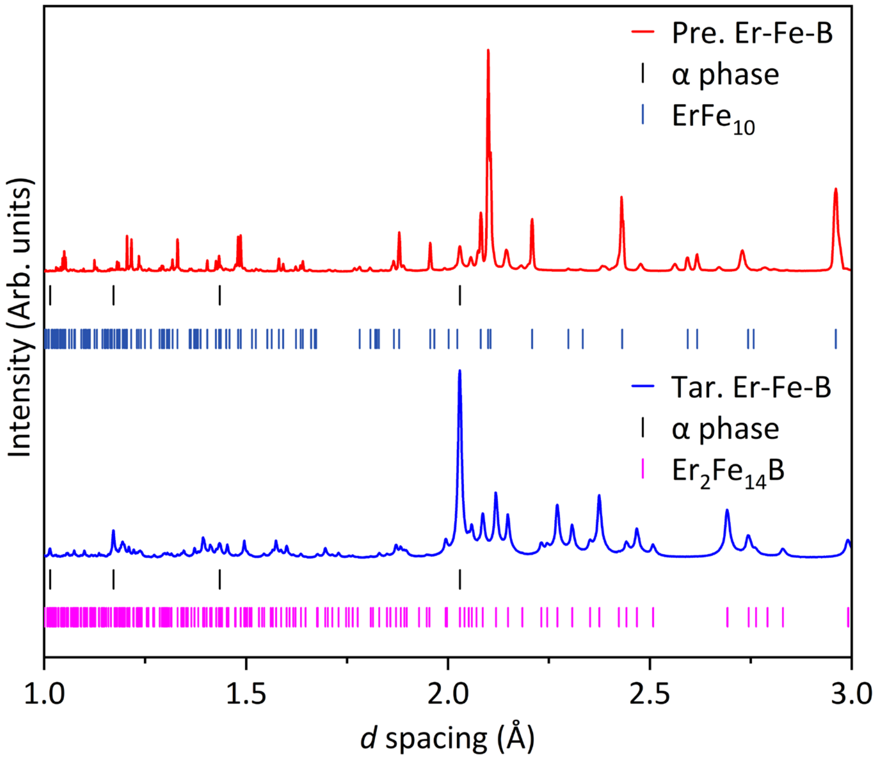

Supplementary Fig. 10 | Comparison of SXRD data between Pre. Er-Fe-B and Tar. Er-Fe-B alloy.

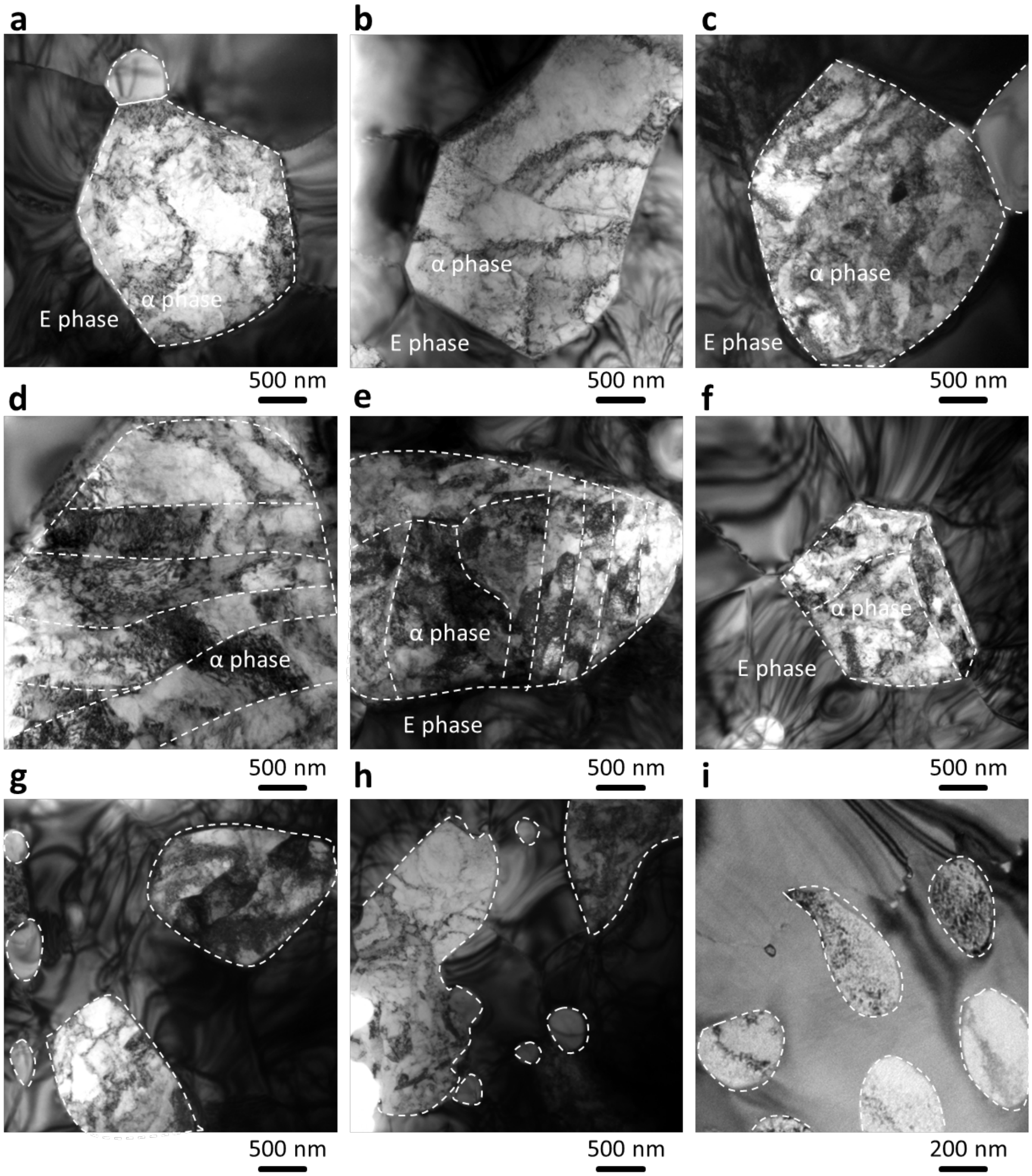

Supplementary Fig. 11 | The microstructure of the re-precipitated α phase is determined by TEM. a-i The morphology of re-precipitated α phases with different grain sizes.

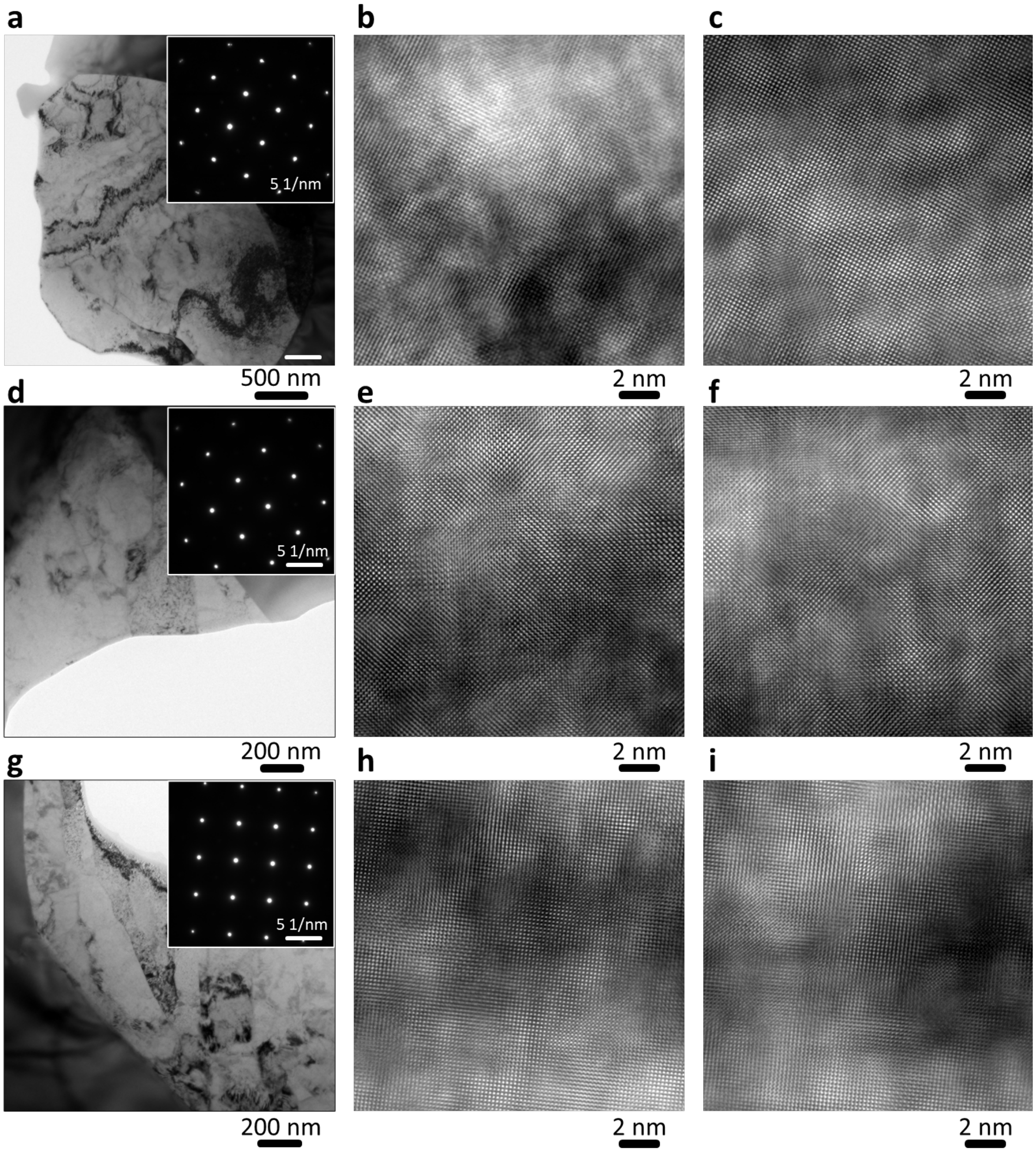

Supplementary Fig. 12 | The microstructure of three different regions re-precipitated α phase. a-c The morphology (**a**) and HRTEM images (**b-c**) of Region 1 along [100] axis inserted the SEAD images. **d-f** The morphology (**d**) and HRTEM images (**e-f**) of Region 2 along [100] axis inserted the SEAD images. **g-i**, The morphology (**g**) and HRTEM images (**h-i**) of Region 3 along [100] axis inserted the SEAD images.

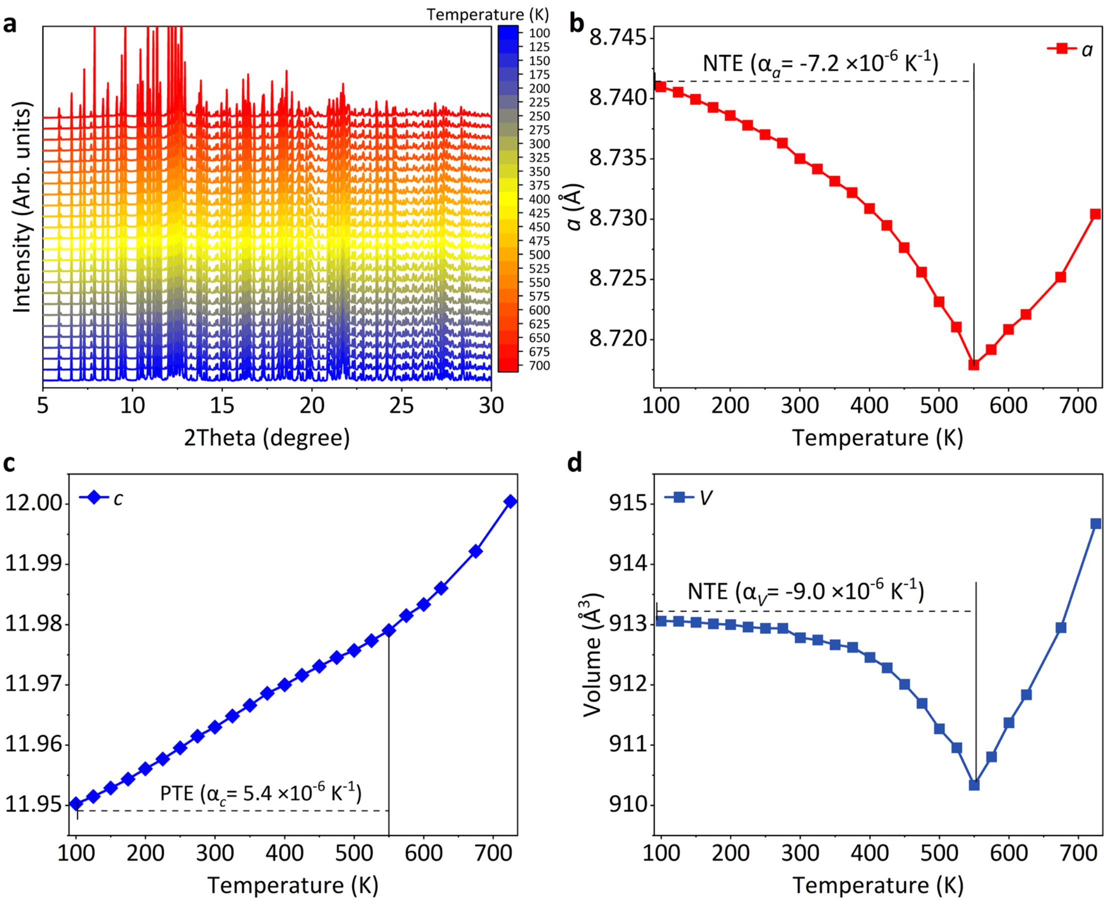

Supplementary Fig. 13 | Lattice thermal expansion of the $Er_2Fe_{14}B$ compound. a Variable temperature synchrotron X-ray radiation diffraction patterns of pure $Er_2Fe_{14}B$ compound. **b-d** The lattice thermal expansion of *a* axis (**b**), *c* axis (**c**), and Volume (**d**).

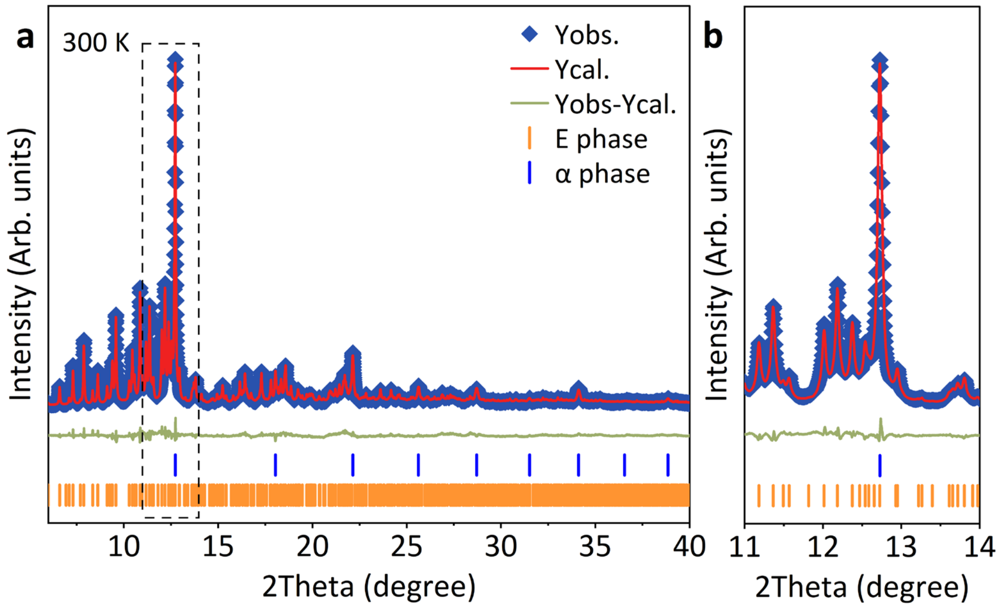

Supplementary Fig. 14 | The Rietveld refinement pattern of the Tar. Er-Fe-B alloy at room temperature (λ = 0.45 Å). a The Rietveld refinement of the Tar. Er-Fe-B alloy. **b** The enlarged profiles are marked in (**a**).

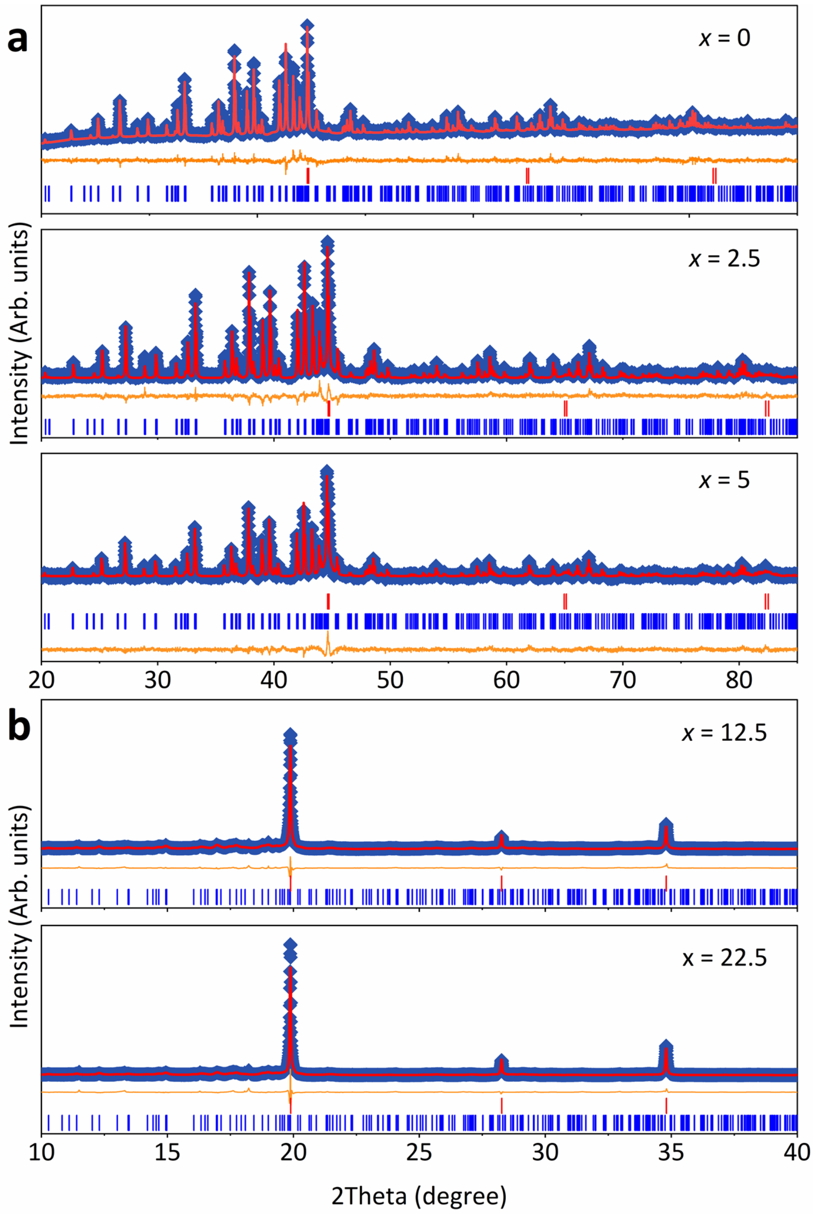

Supplementary Fig. 15 | The X-ray diffraction profiles. a The Rietveld refinement of $x = 0$, $x = 2.5$ and $x = 5$ compositions (XRD, $\lambda = 1.45$ Å). **b** The Rietveld refinement of $x = 12.5$ and $x = 22.5$ compositions (SXRD, $\lambda = 0.70$ Å).

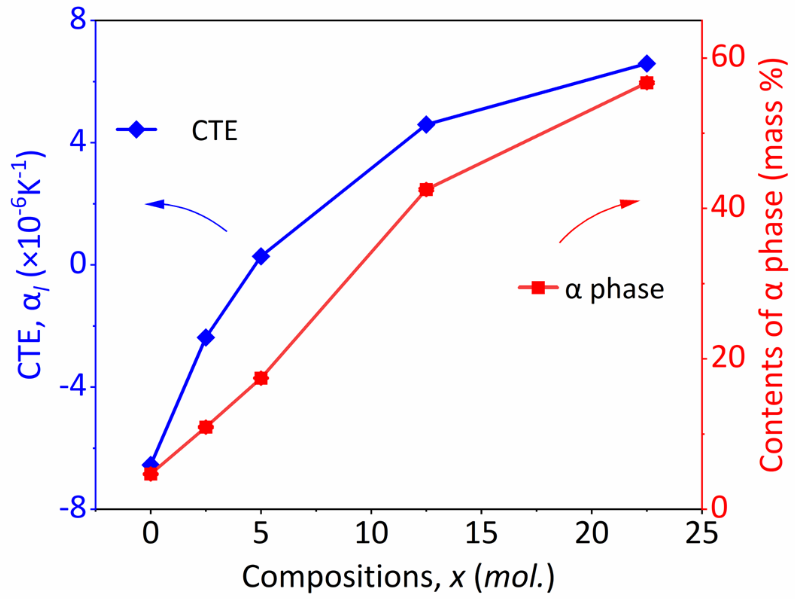

Supplementary Fig. 16 | The comparison of compositions versus CTE and contents of α phase. The error bars donate standard deviation.

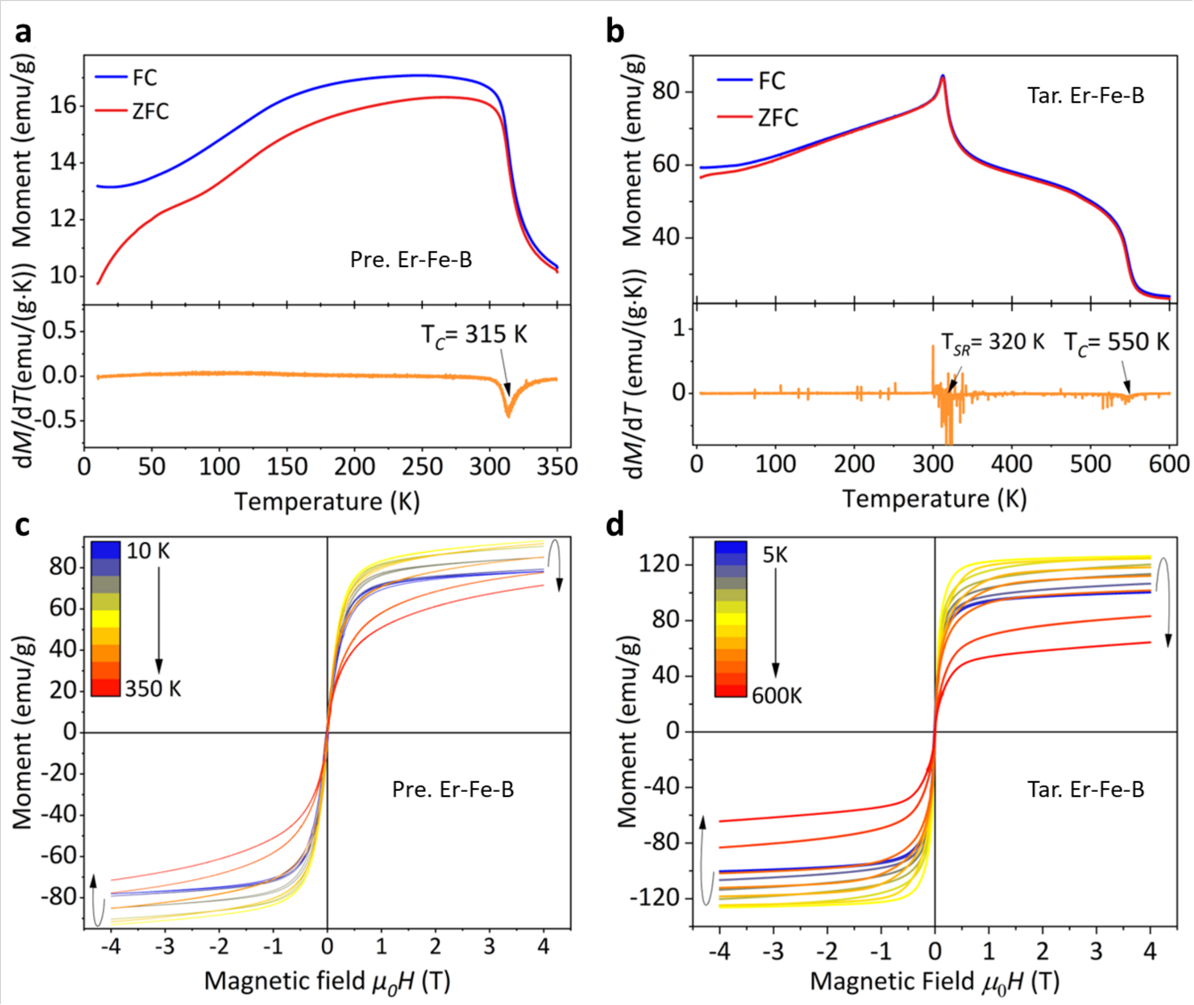

Supplementary Fig. 17 | The magnetization curves of the Pre. Er-Fe-B (ZTE composition) and Tar. Er-Fe-B alloys. a-b Zero-field-cooling (*ZFC*) and field-cooling (*FC*) magnetization (*M*) at a magnetic field of 500 Oe (Pre. Er-Fe-B) and 1000 Oe (Tar. Er-Fe-B), respectively. **c-d** Temperature dependence of magnetization curves (*M-H*) of Pre. Er-Fe-B (**c**) and Tar. Er-Fe-B (**d**) alloys. The Pre. Er-Fe-B exhibits ferrimagnetic behavior below T_C= 315 K, which is consistent with the stoichiometric Er_2Fe_{17} compound. The negative thermal expansion behavior is consistent with magnetic ordering (T_C= 315 K). Both the magnetic ordering and crystallographic texture control the anisotropic thermal expansion performance [17]. The Tar. Er-Fe-B alloy exhibits ferrimagnetic behavior with a spin reorientation at low temperatures (T_{SR}= 320 K). The zero thermal expansion behavior is consistent with magnetic ordering (T_C= 550 K).

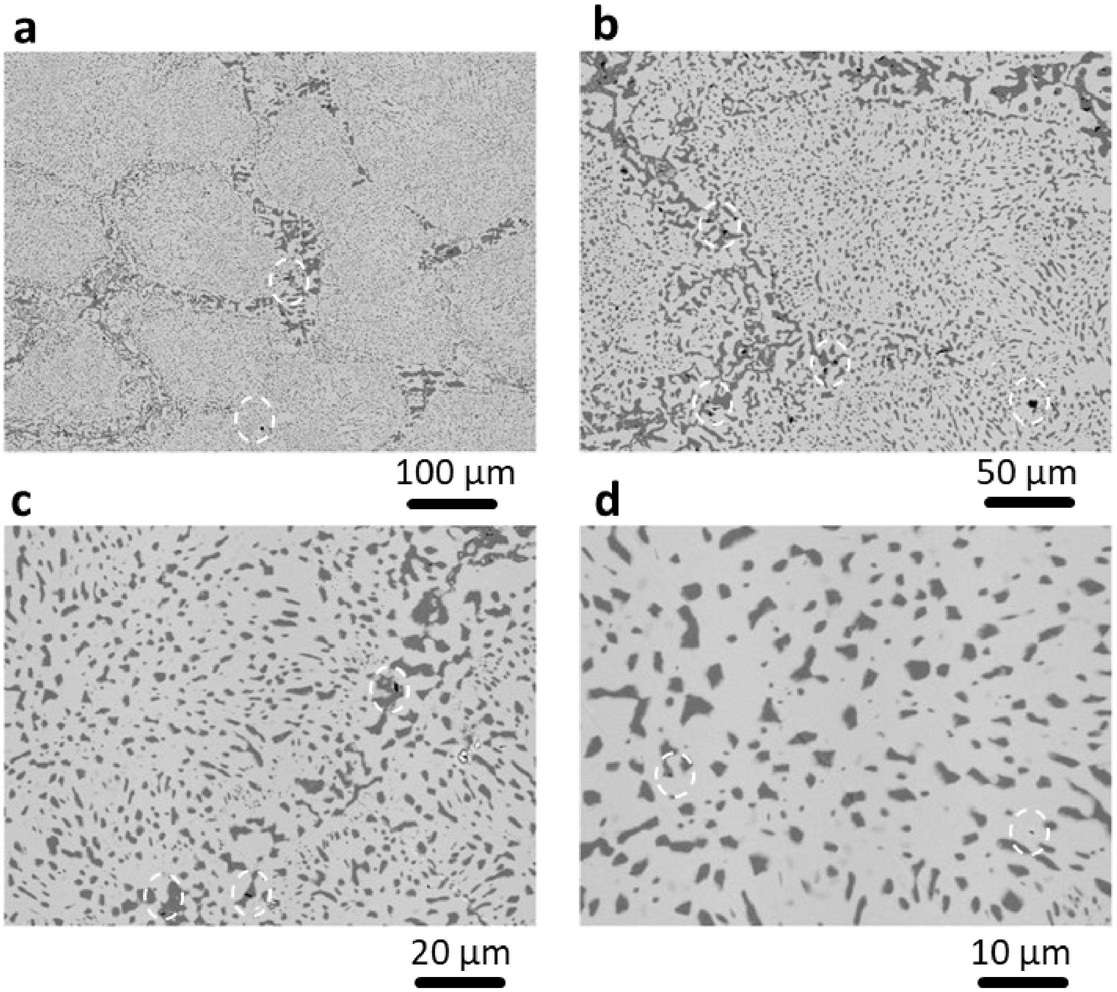

Supplementary Fig. 18 | The microstructure of the Tar. Er-Fe-B after 200th thermal cycles.
a-d The microstructure of Tar. Er-Fe-B at different scales. We did not observe microcracks at the interface, which may be due to the more robust interface of the naturally composited dual-phase alloy. But we observed some shrinkage cavities (marked by white circles), which may have been generated during the synthesis.

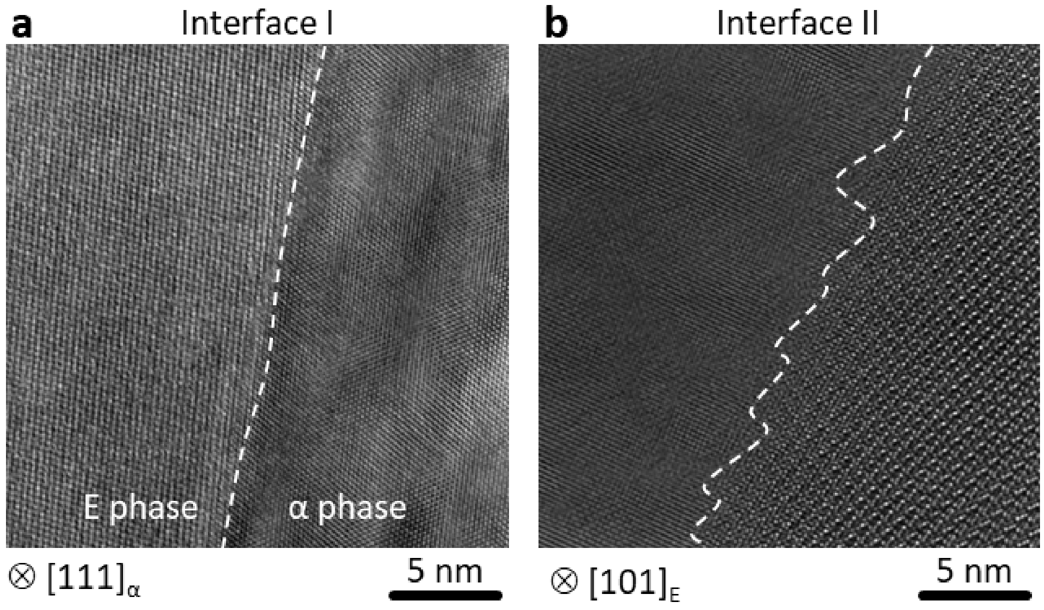

Supplementary Fig. 19 | The two typical phase interfaces of the Tar. Er-Fe-B alloy. a-b The HRTEM image of Region 1 (**a**) and Region 2 (**b**), respectively.

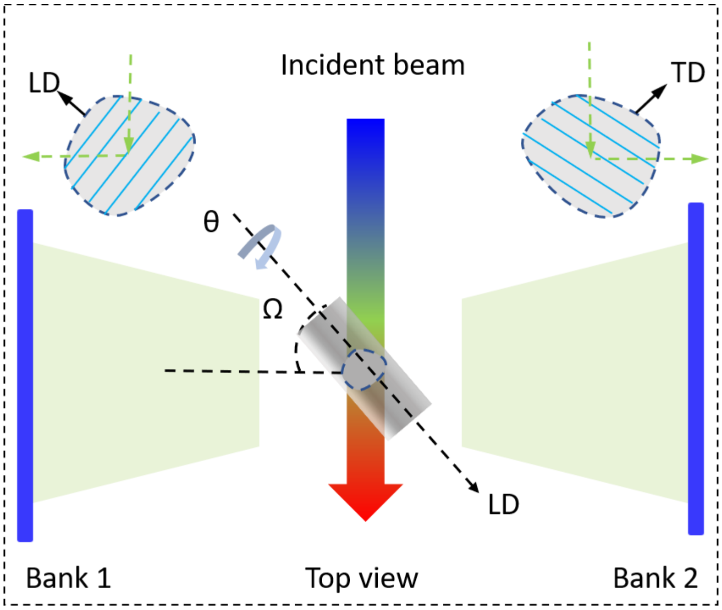

Supplementary Fig. 20 | Schematic diagram of the real-time in-situ neutron diffraction experimental set-up from the top view. The sample is horizontal and positioned at 45° from the incident beam such that Bank 1 probes the strain component along the LD, while Bank 2 simultaneously probes the strain component in the TD, as shown in two insets [7, 21].

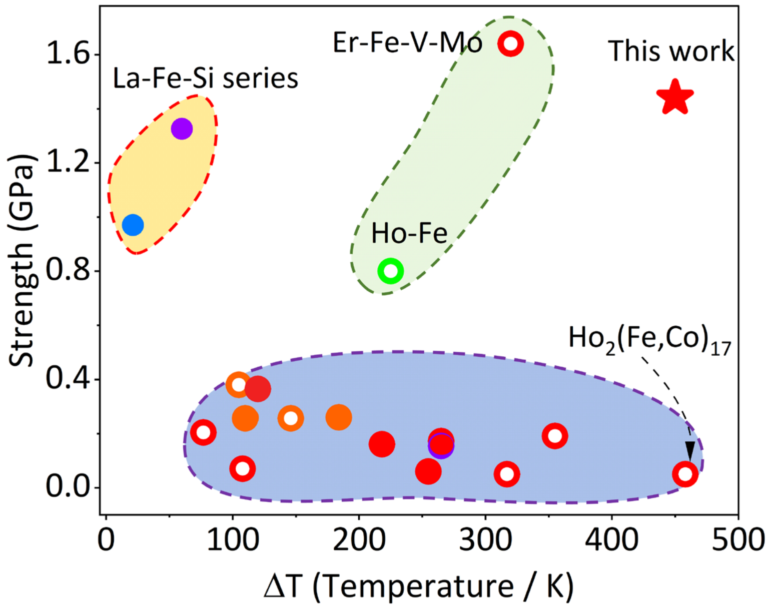

Supplementary Fig. 21 | The summary of compressive strength versus temperature window of typical ZTE alloys. Note: hollow dots represent anisotropic thermal expansion, and solid dots represent isotropic thermal expansion.

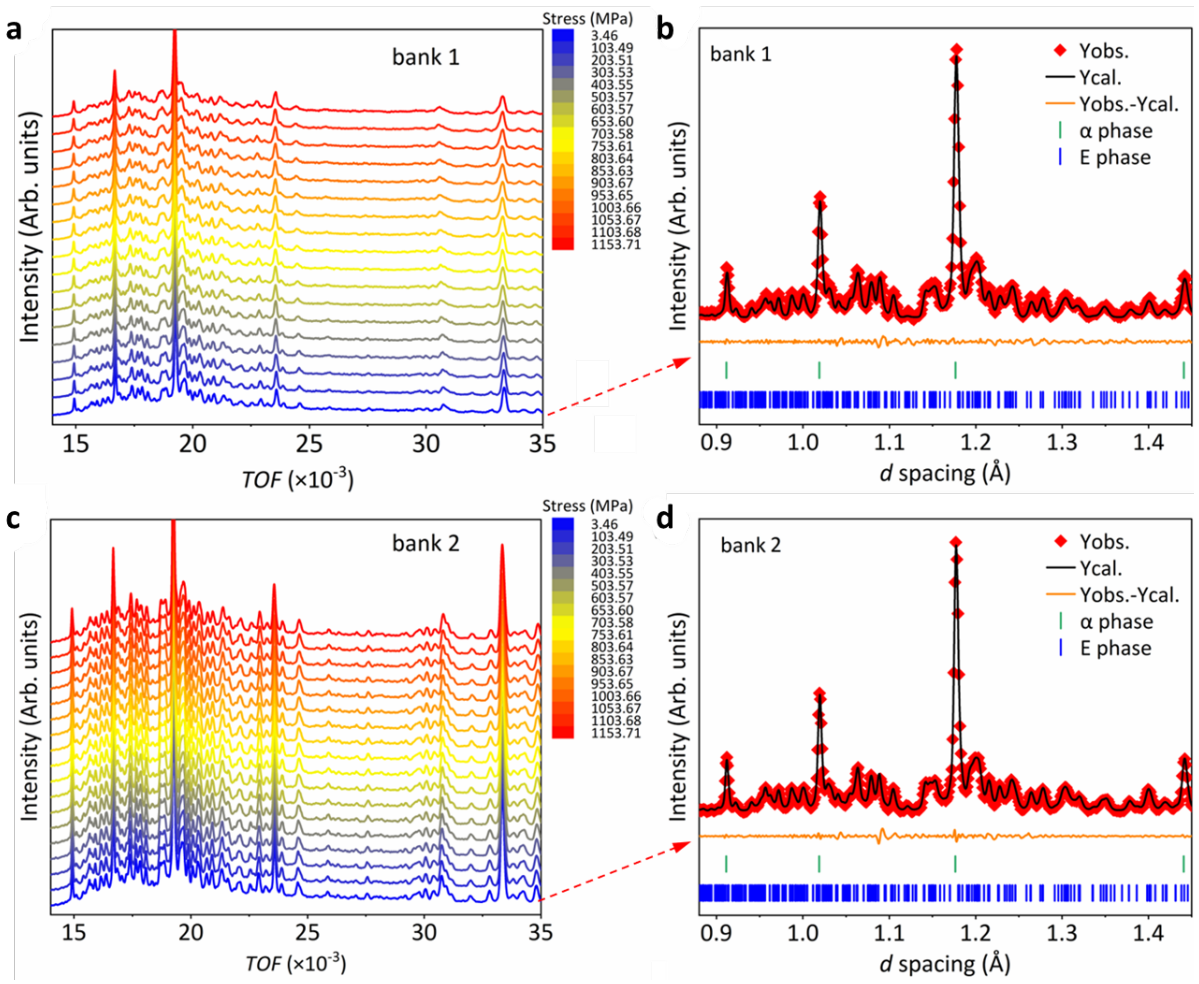

Supplementary Fig. 22 | The in-situ loading neutron diffraction patterns of the Tar. Er-Fe-B were collected by Bank 1 and Bank 2 probes, respectively. a The neutron diffraction patterns along with LD. b) The fitting patterns were collected by the bank 1 probe. **b** The fitting patterns were collected by the bank 2 probe. **c** The neutron diffraction patterns in the TD-ND plane. **d** The Le Bail fitting patterns of the in-situ loading neutron diffraction at 0 MPa.

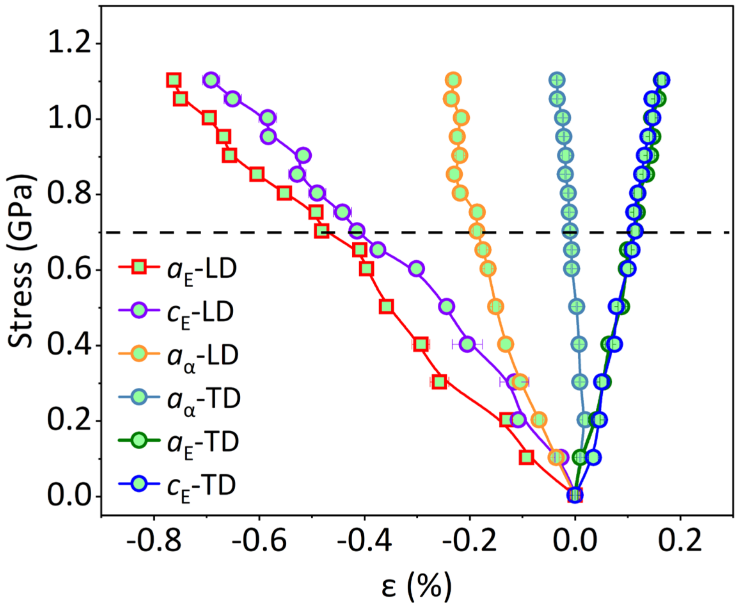

Supplementary Fig. 23 | Lattice evolution of the E and α phase as a function of applied stress determined by full pattern Rietveld refinement. The average lattice strain (ε_i) against applied stress is attained by the Rietveld refinement. The similar trend of lattice strain (single fitting and full-spectrum refinement) further verifies the reasonability of its mechanical behavior and the consistency from local to average lattice strain. Besides, the phase-specific stress in the E and α phase is calculated by the average lattice strain (ε_i). The error bars donate standard deviation.

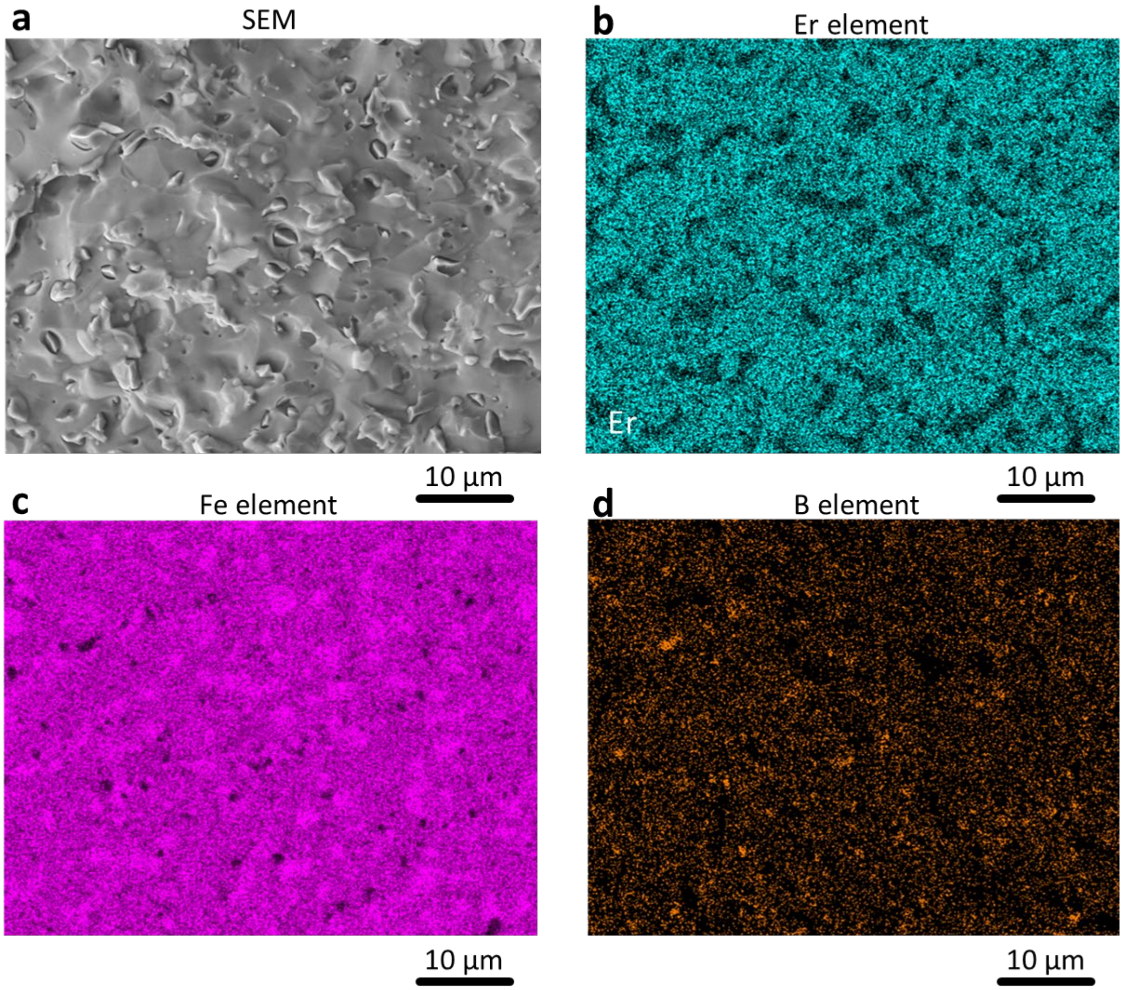

Supplementary Fig. 24 | The microstructure of the fracture surface. a The SEM image of the fracture surface. **b-d** The mapping of the Er (**b**), Fe (**c**), and B (**d**) elements, respectively.

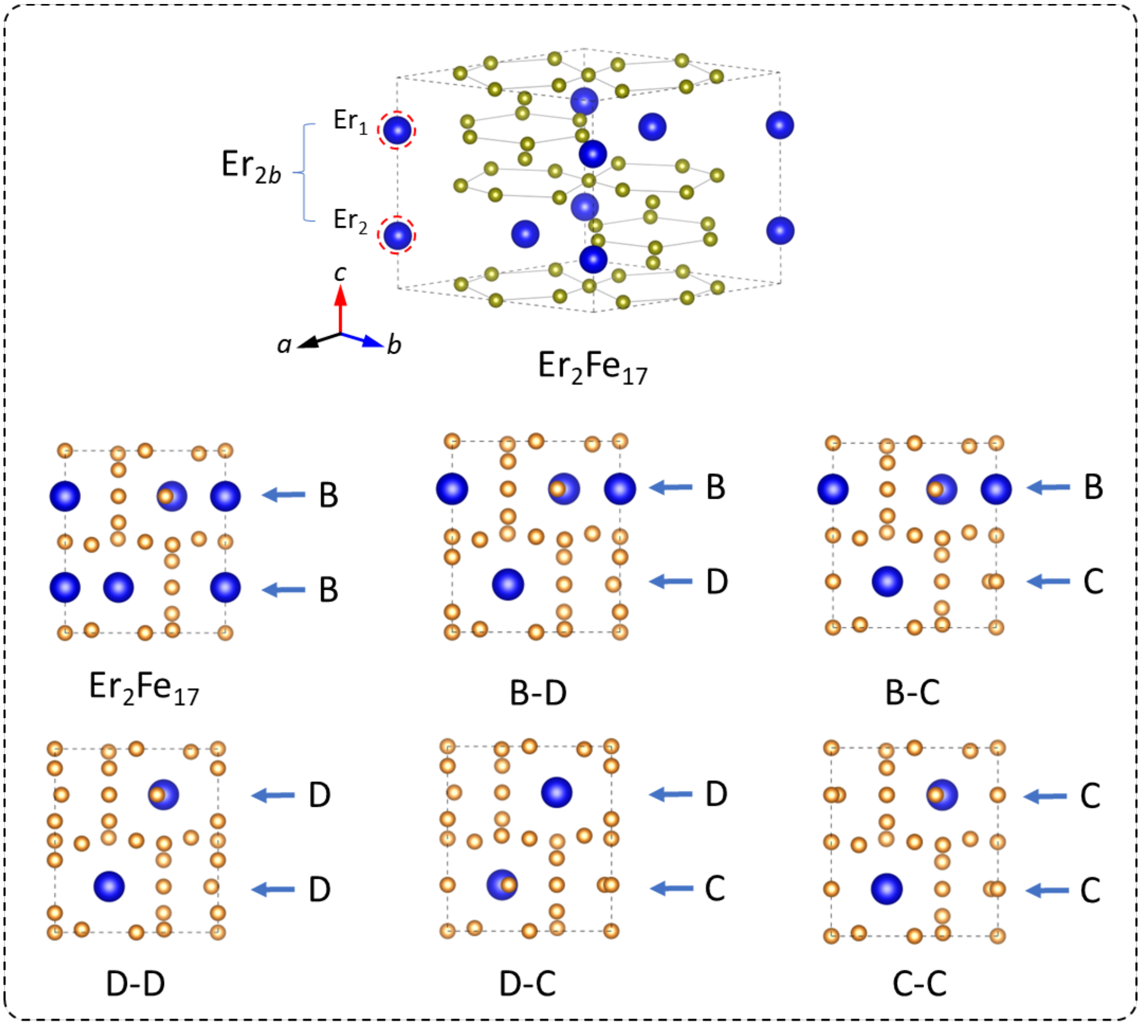

Supplementary Fig. 25 | All possible chemical configurations of this hexagonal lattice.

Supplementary References

1. Song Y., *et al.* Zero thermal expansion in magnetic and metallic Tb(Co,Fe)$_2$ intermetallic compounds. *J. Am. Chem. Soc.* **140**, 602-605 (2018).
2. Dan S., Mukherjee S., Mazumdar C., Ranganathan R. Zero thermal expansion with high Curie temperature in $Ho_2Fe_{16}Cr$ alloy. *RSC Adv.* **6**, 94809-94814 (2016).
3. Li L., Gong Y., Wang C., Zhang Y., Xu F. Achievement of zero thermal expansion covering room temperature in the La(Fe,Al)$_{13}$-based bulks with reduced annealing time. *Physica B* **636**, 413897 (2022).
4. Pang X., Song Y., Shi N., Xu M., Zhou C., Chen J. Design of zero thermal expansion and high thermal conductivity in machinable *x*LFCS/Cu metal matrix composites. *Compos. Part B-Eng.* **238**, 109883 (2022).
5. Li S., Huang R., Zhao Y., Wang W., Han Y., Li L. Zero thermal expansion achieved by an electrolytic hydriding method in La(Fe,Si)$_{13}$ Compounds. *Adv. Funct. Mater.* **27**, 1604195 (2017).
6. Song X., *et al.* Adjustable zero thermal expansion in antiperovskite manganese nitride. *Advanced Materials* **23**, 4690-4694 (2011).
7. Yu C., *et al.* Plastic and low-cost axial zero thermal expansion alloy by a natural dual-phase composite. *Nat. Commun.* **12**, 4701 (2021).
8. Hu J., *et al.* Adjustable magnetic phase transition inducing unusual zero thermal expansion in cubic RCo_2-based intermetallic compounds (R = Rare Earth). *Inorg. Chem.* **58**, 5401-5405 (2019).
9. Hu J. Y., *et al.* A case of multifunctional intermetallic compounds: negative thermal expansion coupling with magnetocaloric effect in (Gd,Ho)(Co,Fe)$_2$. *Inorg. Chem. Front.* **6**, 3146-3151 (2019).
10. Wang J., *et al.* Balancing negative and positive thermal expansion effect in dual-phase La(Fe,Si)$_{13}$/α-Fe in-situ composite with improved compressive strength. *J. Alloy Compd.* **769**, 233-238 (2018).
11. Liu J., *et al.* Realization of zero thermal expansion in La(Fe,Si)$_{13}$ -based system with high mechanical stability. *Mater. Design* **148**, 71-77 (2018).
12. Cao Y., *et al.* Ultrawide temperature range super-Invar behavior of R_2(Fe,Co)$_{17}$ materials (R = Rare Earth). *Phys. Rev. Lett.* **127**, 055501 (2021).
13. Lin K., *et al.* High performance and low thermal expansion in Er-Fe-V-Mo dual-phase alloys. *Acta Mater.* **198**, 271-280 (2020).
14. Huang R., *et al.* Giant negative thermal expansion in $NaZn_{13}$-type La(Fe, Si, Co)$_{13}$ compounds. *J. Am. Chem. Soc.* **135**, 11469-11472 (2013).
15. Song Y., *et al.* Opposite Thermal Expansion in Isostructural Noncollinear Antiferromagnetic Compounds of Mn_3A (A = Ge and Sn). *Chem. Mater.* **30**, 6236-6241 (2018).
16. Li L., *et al.* Good comprehensive performance of Laves phase $Hf_{1-x}Ta_xFe_2$ as negative thermal expansion materials. *Acta Mater.* **161**, 258-265 (2018).
17. Qiao Y., *et al.* Controllable thermal expansion and magnetic structure in Er_2(Fe,Co)$_{14}$B intermetallic compounds. *Inorg. Chem. Front.* **6**, 3225-3229 (2019).
18. Zhao Y., *et al.* Giant negative thermal expansion in bonded MnCoGe-based compounds with Ni_2In-type hexagonal structure. *J. Am. Chem. Soc.* **137**, 1746-1749 (2015).
19. Van Ende M.-A., Jung I.-H. Critical thermodynamic evaluation and optimization of the Fe–B, Fe–Nd, B–Nd and Nd–Fe–B systems. *J. Alloy Compd.* **548**, 133-154 (2013).

20. Cao Y., *et al.* Role of "Dumbbell" pairs of Fe in spin alignments and negative thermal expansion of Lu_2Fe_{17}-based intermetallic compounds. *Inorganic Chemistry* **59**, 11228-11232 (2020).
21. Beese A., Wang Z., Stoica A., Ma D. Absence of dynamic strain aging in an additively manufactured nickel-base superalloy. *Nat. Commun.* **9**, 2083 (2018).
